# Enhancement of Microbial Biodesulfurization via Genetic Engineering and Adaptive Evolution

**DOI:** 10.1371/journal.pone.0168833

**Published:** 2017-01-06

**Authors:** Jia Wang, Robert R. Butler, Fan Wu, Jean-François Pombert, John J. Kilbane, Benjamin C. Stark

**Affiliations:** Department of Biology, Illinois Institute of Technology, Chicago IL, United States of America; Tallinn University of Technology, ESTONIA

## Abstract

In previous work from our laboratories a synthetic gene encoding a peptide (“Sulpeptide 1” or “S1”) with a high proportion of methionine and cysteine residues had been designed to act as a sulfur sink and was inserted into the *dsz* (desulfurization) operon of *Rhodococcus erythropolis* IGTS8. In the work described here this construct (*dszAS1BC*) and the intact *dsz* operon (*dszABC*) cloned into vector pRESX under control of the (*Rhodococcus*) *kstD* promoter were transformed into the desulfurization-negative strain CW25 of *Rhodococcus qingshengii*. The resulting strains (CW25[pRESX-*dszABC*] and CW25[pRESX-*dszAS1BC*]) were subjected to adaptive selection by repeated passages at log phase (up to 100 times) in minimal medium with dibenzothiophene (DBT) as sole sulfur source. For both strains DBT metabolism peaked early in the selection process and then decreased, eventually averaging four times that of the initial transformed cells; the maximum specific activity achieved by CW25[pRESX-*dszAS1BC*] exceeded that of CW25[pRESX-*dszABC*]. Growth rates increased by 7-fold (CW25[pRESX-*dszABC*]) and 13-fold (CW25[pRESX-*dszAS1BC*]) and these increases were stable. The adaptations of CW25[pRESX-*dszAS1BC*] were correlated with a 3-5X increase in plasmid copy numbers from those of the initial transformed cells; whole genome sequencing indicated that during its selection processes no mutations occurred to any of the *dsz*, S1, or other genes and promoters involved in sulfur metabolism, stress response, or DNA methylation, and that the effect of the sulfur sink produced by S1 is likely very small compared to the cells’ overall cysteine and methionine requirements. Nevertheless, a combination of genetic engineering using sulfur sinks and increasing Dsz capability with adaptive selection may be a viable strategy to increase biodesulfurization ability.

## Introduction

Biological removal of organic sulfur from petroleum has been investigated for more than twenty-five years as a possible cost effective and environmentally benign alternative to chemical removal strategies [1]. While the removal of sulfur from organic compounds in petroleum can be accomplished by chemical engineering processes such as hydrodesulfurization [2], oxidative desulfurization [3,4], and extractive desulfurization [5], the removal of sulfur to the increasingly stringent levels required by environmental regulations is challenging. This is especially true of dibenzothiophene (DBT) and its alkylated derivatives (the most common organic sulfur compounds in petroleum, particularly in heavy fractions) which are recalcitrant to chemical desulfurization [2,6,7]. The specificity of biochemical reactions and the ability to achieve desulfurization at ambient temperatures and pressures, and without the need to supply hydrogen, has resulted in the continued interest in biodesulfurization [8]. The first desulfurization pathway characterized (the “4S pathway” mediated by enzymes DszA, DszB and DszC, encoded by the *dszABC* operon) is that from *Rhodococcus erythropolis* strain IGTS8 [1,9–12]. The 4S pathway removes the sulfur from DBT while maintaining its caloric value. Currently available desulfurization biocatalysts, however, are not suitable for practical applications, as these would require large increases in enzyme activity [8] as well as more thermostable enzymes and hosts [13,14].

Analogs of the IGTS8 *dszABC* operon encoding enzymes that function above 50°C have been characterized [15–20], and a great deal of work has been done to use modern genetic technology to enhance enzyme levels [21,22]. However, prior research concerning the biodesulfurization trait has demonstrated that attempts to overexpress the genes that encode the biochemical pathway for the sulfur-specific cleavage of carbon-sulfur bonds, *dszABC*, failed to yield dramatic improvements in desulfurization activity, and seem to have reached a ceiling that is far below the required level for an economical oil biodesulfurization process [8]. Moreover, the involvement of genes other than the *dszABC* genes in the optimal expression of the desulfurization trait has been demonstrated, suggesting that yet to be identified genes may contribute to the optimal expression of the biodesulfurization trait [23]. As a new approach, a synthetic gene *S1* encoding a methionine and cysteine rich peptide (Sulpeptide 1) to act as a sulfur sink was designed and inserted into the *dszABC* operon (producing *dszAS1BC*), in a manner that would force its expression along with that of the *dszA*, *B*, and *C* genes. The hope was to produce a loop in which increasing expression of S1 would lead to increased *dszABC* expression which would lead to even greater S1 expression and so on [24].

After transforming recombinant plasmids containing either the *dszABC* or *dszAS1BC* constructs into desulfurization-negative *Rhodococcus opacus*, both recombinants were subjected to adaptive selection, in which cells were grown in chemically defined medium (CDM) with DBT as the sole sulfur source. Cultures were transferred into fresh CDM-DBT when growth reached the midpoint of log phase, so as to select for the fastest growing (and presumably fastest DBT metabolizing) strains. The idea was to combine this adaptive selection with the forced expression of *S1* (to continually increase sulfur demand) in an effort to breach the ceiling for the expression of the Dsz enzymes. The extent of the first selection experiment (40 passages) served as proof of concept that this approach might succeed; adaptive selection in passages 30 through 40 led to a large increase in desulfurization activity, although the presence of the sulpeptide was correlated with a much smaller increase [24].

The experiments reported here constitute a more extensive and comprehensive continuation of this work. The desulfurization negative microbial host *Rhodococcus qingshengii* CW25 was selected for use in this study rather than a desulfurization competent host such as *Rhodococcus erythropolis* IGTS8 because the use of a *dsz*-negative culture allowed for the selection of the *dsz* trait in transformation experiments (specifically, we wished to compare strains both with and without expression of the sulpeptide, and the sulpeptide containing operon could only be constructed in vitro), and to better enable the detection of non-*dsz* genes that may contribute to the functioning of the biodesulfurization trait. We reasoned that mutations in non-*dsz* genes that influence the biodesulfurization trait, and may already be present in *R*. *erythropolis* IGTS8, would be more readily detected in the non-desulfurizing culture *R*. *qingshengii* CW25. We chose not to alter the expression of genes such as *dszD* that are known to influence the desulfurization trait [14] because we sought to use adaptive evolution to learn how to prioritize genetic/biochemical targets for future research.

Our results indicate that improved efficiency of the utilization of sulfur and increases in the copy numbers of the *dsz* operon, without any other mutation, are sufficient to account for the increases in desulfurization that occurred during selection. The presence of the sulpeptide gene, however, does not drive the reaction faster, as anticipated, but rather enhances growth of the engineered bacteria, and appears to be responsible for augmenting their overall biodesulfurization efficiency. Although it was designed with a signal sequence to mediate its secretion from the cell, the sulpeptide protein may act as a sulfur reserve that prevents bacterial growth slowdowns when sulfur in the environment becomes scarce, which suggests that the use of sulfur sinks in combination with multiple copies of the *dsz* operon is an engineering strategy that may help the overall efficiency of biodesulfurization.

## Materials and Methods

### Bacterial strains, plasmids and media

*Rhodococcus qingshengii* CW25, which is desulfurization negative, was used as a host for expression of the desulfurization operon (*dszABC*) from *Rhodococcus erythropolis* IGTS8; strain CW25 has been deposited as NRRL B-65406 in the ARS Culture Collection (NRRL) of the USDA in Peoria, Illinois (USA). We had previously constructed plasmids pRESX-*dszABC* and pRESX-*dszAS1BC* [24]; they contain the native *dszABC* operon or its sulpeptide-containing derivative, respectively, cloned into the *E*. *coli*-*Rhodococcus* shuttle vector pRESX [25] under control of the *kstD* promoter (P*kstD*).

For most experiments testing desulfurization ability, cells were grown in CDM medium, prepared as described by Wang et al. [26], but without vitamins or yeast extract; kanamycin was also omitted from this medium to eliminate any sulfur contributed by its sulfate salt. To determine the minimal concentration of sulfur source required for maximum growth rate, CDM medium without kanamycin was made with either sulfate (ranging from 0–1 mM) or DBT (ranging from 0–0.5 mM) serving as the sole sources of sulfur; glucose (0.06 M) was the carbon source and growth was monitored by OD_600nm_. The solubility of DBT in water is about 6 μM [27], so that DBT at minimal concentrations of 0.05 mM and above (see also below) replenished the soluble DBT as it was utilized by the cells.

### Transformation of pRESX-dszABC and pRESX-dszAS1BC into strain CW25

CW25 cells were grown in LB medium at 30°C, 200 rpm to an OD600nm of 0.4–0.5. Ampicillin was then added to the culture to a final concentration of 200 μg/ml (to weaken the cell wall) followed by 2 more hours at 30°C, 200 rpm. Cells were harvested by centrifugation, washed 4 times with ice cold 0.3 M sucrose, and resuspended in 1 ml of ice cold 0.5 M sucrose.

30 μl of cells prepared in this way were mixed with 500 ng of either pRESX-*dszABC* or pRESX-*dszAS1BC*, transferred to an ice-cold electroporation cuvette (1 mm gap), and subjected to a single pulse at 1500 V, 200 μs. This was followed immediately by dilution with 1 ml of pre-warmed SOC medium [28] followed by incubation for 6 hours at 30°C, 200 rpm. Cells were harvested by centrifugation, resuspended in 50 μl of SOC, and spread on LB plates containing 500 μg/ml kanamycin. Random colonies from these plates were selected and confirmed by colony PCR using primers specific for P*kstD* (to identify pRESX) and *S1* (diagnostic for the *dszAS1BC* construct).

### Adaptive selection

From LB-kanamycin (500 μg/ml) plate stocks a colony of each transformant (CW25[pRESX-*dszABC*] and CW25[pRESX-*dszAS1BC*]; denoted as “P0” in each case) was inoculated into CDM-0.05 mM DBT with either ethanol (0.043 M) or glucose (0.06 M) as the sole carbon source and grown at 30°C, 200 rpm. Kanamycin (sulfate) was omitted from this medium, with plasmid stability forced by the need to retain the *dszABC* operon. Cultures were monitored spectrophotometrically during incubation, and were passaged (subcultured) into fresh medium when growth reached the middle of log phase. Thus, this protocol selected for the cells able to grow the fastest with DBT as sole sulfur source. Each passage averaged about 4 generations.

Three individual selection experiments, with some variation in parameters, were performed (Table 1). At intervals, cultures were plated onto LB-kanamycin (500 μg/ml) plates to confirm culture purity. In the first experiment, a single colony was selected at intervals for determination of plasmid stability using colony PCR as described above, grown and assayed for DszABC specific activity, and also used as the inoculum for the downstream passage. In the second and third experiments, three single colonies were selected randomly at each interval for PCR and specific activity assay, and, to lessen the possibility of variations resulting from random selection, the one with the highest specific activity was the inoculum for the downstream passage. Passage numbers are denoted with “P” and the passage number.

**Table 1:** Details of the three selection experiments.

Experiment No.	Strains	Medium	No. of Passages (P)	Inoculum
1	CW25[pRESX-*dszABC*] CW25[pRESX-*dszAS1BC*]	CDM-DBT (0.05 mM)-glucose (0.06 M)	100	Initial transformants (P0)
2	CW25[pRESX-*dszABC*] CW25[pRESX-*dszAS1BC*]	CDM-DBT (0.05 mM)-ethanol (0.043 M)	30	P4 from expt. 1 for both strains
3	CW25[pRESX-*dszAS1BC*]	CDM-DBT (0.05 mM)-glucose (0.06 M)	30	Initial transformant (P0)

### Specific activity (“resting cell”) assay

Single colonies of CW25 transformants (or strain IGTS8 as positive control) were grown in kanamycin-free CDM-0.05 mM DBT with either glucose (0.06 M) or ethanol (0.043 M) as the carbon source. These media compositions were determined to be optimal for DszABC activity through a preliminary series of experiments testing various concentrations of glucose and ethanol, two basal media, and DBT compared to DMSO as sole sulfur source (see Results and S1 Fig). Cells were grown at 30°C, 200 rpm to mid-log phase (OD_600nm_ of about 0.4–0.7); this pre-culture was inoculated into 50 ml of the same medium in a 250 ml flask with initial OD_600nm_ of no more than 0.05, and grown at 30°C, 200 rpm to mid-log phase (OD_600nm_ of about 0.4–0.7).

After harvesting by centrifugation and washing once with CDM containing no DBT (or ethanol, glucose, or kanamycin), cells were resuspended in the same CDM, with OD_600nm_ adjusted to 1.0. 5 ml of the cell suspension was transferred to a 25 ml flask for the desulfurization resting cell activity assay (which measures conversion of DBT to 2-hydroxybiphenyl (2-HBP) by the Gibbs assay; [24]). 25 μl of 400 mM DBT (dissolved in ethanol) was added, to make a starting DBT concentration of 2 mM. Data points were taken at 0, 1, 2 and 4 hours.

Cells from the rest of each suspension were harvested, washed with, and then resuspended in, distilled water and then transferred to a preweighed aluminum dish for drying and dry cell weight (DCW) determination. The slope of the plot of μmol 2-HBP /g-DCW as a function of time was the desulfurization rate; one unit is defined as 1 μmol of 2-HBP produced by 1 g dry cell weight of cells in 1 hour.

### Desulfurization ability of growing cells

Pre-cultures were prepared as described above for specific activity measurements, and inoculated into 50 ml of fresh kanamycin-free CDM-0.05 mM DBT containing 0.043 M ethanol in 250 ml flasks with an initial OD_600nm_ of no more than 0.05. Growth conditions were 30°C, 200 rpm. When the culture reached late log phase (OD_600nm_ of about 0.7–1.0), the time was recorded and 1 ml was taken for Gibbs assay of the 2-HBP accumulated in the growth medium.

### Cell doubling time determinations

Pre-cultures were prepared as described above, inoculated into 50 ml of fresh kanamycin-free CDM-0.05 mM DBT containing 0.043 M ethanol in 250 ml flasks, and grown at 30°C, 200 rpm. The initial OD_600nm_ was 0.05. OD_600nm_ was read multiple times until the end of log phase. Data of OD_600nm_ were converted into log_10_OD_600nm_ values, which were plotted against incubation time (in hours), and the maximum slope (determined with at least 3 time points) used to calculate cell doubling time.

### Genomic sequencing, assembly and annotation

To sample genetic changes on a genome-wide scale that may have occurred during adaptive selection, DNA was purified from four selected passages of strain CW25[pRESX-*dszAS1BC*], including P0, P4 and P100 from the first selection experiment and P10 from the third selection experiment, using the Power Soil DNA extraction kit (MoBio, Carlsbad, CA, USA). Illumina sequencing was conducted on a MiSeq sequencer (Illumina, San Diego, CA, USA) at Université Laval’s Plate-forme d’Analyses Génomiques (Québec, QC, Canada). A 300-bp paired-end protocol was run on TruSeq (Illumina) DNA libraries (insert sizes: P0 514 ± 117 bp; P4 506 ± 115 bp; P10 425 ± 118 bp; P100 445 ± 126 bp).

Genome assembly was completed utilizing four *de novo* assemblers: SPAdes 3.5 [29]; Ray 2.3.1 [30]; A5-miseq 20141120 [31]; and Geneious 7.1 [32]. For the Ray assemblies, kmer size was determined using Kmergenie 1.6663 [33]. For Geneious assemblies, paired-end reads were trimmed for quality with an Error Probability Limit of 0.001 and *de novo* assembled at medium sensitivity using only paired reads. Contigs smaller than 1 kbp were discarded, and assemblies were compared using Quast 2.3 [34]. For each genome, the best assembly was selected: P0, SPAdes (24 contigs, 6,325,434 bp total length, 2,642,655 bp longest contig, 1,373,508 bp N_50_); P4, Geneious (18 contigs, 6,259,093 bp total length, 1,119,966 bp longest contig, 748,535 bp N_50_); P10, SPAdes (25 contigs, 6,402,155 bp total length, 1,640,192 bp longest contig, 696,102 bp N_50_).

The P100 genome assembly was more completely assembled for submission to NCBI as the complete sequence of *Rhodococcus qingshengii* CW25 without the pRESX plasmid (BioProject PRJNA316739). Contigs from the best P100 assembly (Ray, 20 contigs, 6,417,168 bp total length, 1,640,764 bp longest contig, 704,582 bp N_50_) were compared to the other assemblies (using Geneious), and merged in instances where at least two of three other assemblies spanned the gap. The original paired-end reads were then mapped to the merged assembly to verify even and complete coverage at each merger junction. The final P100 assembly contained 10 contigs, 6,399,510 bp total length, 2,298,790 bp longest contig and 1,403,103 bp N_50_, not including the pRESX-*dszAS1BC* plasmid.

Draft annotation of the four assemblies was completed using Prokka 1.11 [35] with a minimum e-value threshold of 1e-30. Final annotation of the P100 assembly of *Rhodococcus qingshengii* CW25 was completed using the NCBI Prokaryotic Genome Annotation Pipeline [36] (Genome Accession LVXC00000000). The original taxonomic classification of this sample was *Rhodococcus erythropolis* CW25; however, NCBI genome submission staff completed an average nucleotide identity (ANI) analysis and concluded this strain was likely misidentified. The strain was found to share 98.12% identity over 93% of the submitted genome sequence to the type genome of *Rhodococcus qingshengii* compared to a lower maximum match of 95.5% to the *Rhodococcus erythropolis* genomes present in the NCBI database (The GenBank Submissions Staff, personal communication, April 1, 2016).

### Genome sequence analysis

The direct comparison of protein sequences in the four passages was accomplished via BLAST protein homology searches [37]. Proteins involved in various sulfur metabolism pathways in other species [38] were searched against the four passage genomes with a minimum e-value threshold of 1e-10 to identify any potential homologs in *Rhodococcus qingshengii* CW25 (Table 2).

**Table 2:** Genes compared among the various CW25[pRESX-*dszAS1BC*] passages.

**4S desulfurization pathway (*Rhodococcus erythropolis*)**
(*dszA*)
(*dszB*)
(*dszC*)
Sulpeptide 1 (*S1*)
**Pathway: methionine biosynthesis I (*Escherichia coli* K12)**
O-succinylhomoserine(thiol)-lyase/O-succinylhomoserine lyase (*metB*) (e-value = 4e-90)
bifunctional β-cystathionase, PLP-dependent and regulator of maltose regulon (*malY*) (no hits)
cystathionine-β-lyase/L-cysteine desulfhydrase (*metC*) (e-value = 2e-39)
cobalamin-independent homocysteine transmethylase (*metE*) (e-value = 0.0)
cobalamin-dependent methionine synthase (*metH*) (e-value = 2e-166)
**Pathway: methionine biosynthesis III (*Corynebacterium glutamicum*)**
homoserine O-acetyltransferase (*metX*) (e-value = 2e-120)
O-acetylhomoserine aminocarboxypropyltransferase (*metY*) (e-value = 4e-175)
**Coenzyme A biosynthesis pathway (*Escherichia coli* K12)**
fused 4'-phosphopantothenoylcysteine decarboxylase and phosphopantothenoylcysteine synthetase (*dfp*) (e-value = 6e-79)
pantetheine-phosphate adenylyltransferase (*coaD*) (e-value = 9e-47)
dephospho-CoA kinase (*coaE*) (e-value = 9e-40)
**Cysteine biosynthesis I (*Escherichia coli* K12)**
serine acetyltransferase (*cysE*) (e-value = 2e-34)
cysteine synthase B (*cysM*) (e-value = 4e-57)
O-acetylserine sulfhydrylase A (*cysK*) (e-value = 3e-92)
**Pathway: sulfate reduction I (*Escherichia coli* K12)**
sulfate adenylyltransferase (*cysD*) (e-value = 4e-157)
sulfate adenylyltransferase (*cysN*) (e-value = 5e-160)
adenylylsulfate kinase (*cysC*) (e-value = 3e-69)
3'-phospho-adenylylsulfate reductase (*cysH*) (e-value = 7e-31)
**Sulfite metabolism I (*Escherichia coli* K12)**
sulfite reductase, flavoprotein subunit (*cysJ*) (e-value = 7e-147)
sulfite reductase, hemoprotein subunit (*cysI*) (no hits)
**Sulfite oxidoreductase (*Rhodococcus erythropolis* PR4)**
putative sulfite oxidase (RER_39830) (no hits)
**Sulfite oxidation I (*Starkeya novella*)**
sulfite oxidoreductase (*sorA*) (no hits)
sulfite oxidoreductase (*sorB*) (no hits)
**Sulfite oxidation II (*Thiobacillus denitrificans*)**
APS reductase alpha subunit (*aprA*) (no hits)
APS reductase beta subunit (*aprB*) (no hits)
adenylylsulfate:phosphate adenylyltransferase (*apt*) (e-value = 3e-16)
**Sulfite oxidation III (*Allochromatium vinosum*)**
adenylylsulfate reductase alpha subunit (*aprA*) (no hits)
adenylylsulfate reductase beta subunit (*aprB*) (no hits)
adenylylsulfate reductase membrane anchor (*aprM*) (no hits)
sulfate adenylyltransferase subunit (*sat*) (no hits)
**Sulfite oxidation V (*Allochromatium vinosum*)**
sulfite dehydrogenase subunit SoeA (*soeA*) (e-value = 4e-13)
sulfite dehydrogenase subunit SoeB (*soeB*) (no hits)
sulfite dehydrogenase subunit SoeC (*soeC*) (no hits)
**Sulfite exporter**
*tauE/safE* (annotated by Prokka)
**Dam methylase**
dam methylase (*dam*) (annotated by Prokka)

Species names in parentheses in the heading of each section are those which supplied the sequences of the genes in that section, against which the genomes of passages P0, P4, P10, and P100 were compared. The sequences of *dszA*, *dszB*, *dszC*, and *S1* are identical to those reported elsewhere [10,24]. E-values for the highest similarity blastp hit in these passages are displayed, except for genes annotated by Prokka, which have a minimum e-value of 1e-30. Genes without blastp matches above e-value 1e-10 are designated as “no hits”.

The coverage of each of the four assemblies and their respective plasmids was determined by read mapping in Geneious and the relative plasmid copy numbers approximated from the plasmid:genome coverage ratio. To detect potential mutations accumulating throughout the generations, the sequence reads from each passage were filtered using a minimum quality score of 32 and a minimum length post-trimming of 150 nt with sickle (https://github.com/najoshi/sickle), mapped on the curated P100 assembly with BWA-SW 0.7.13-r1126 [39], converted from SAM to BAM format with SAMtools 1.3.1 [40], and single nucleotide polymorphisms (SNPs) and insertions-deletions (indels) calculated using VarScan 2.4.2 [41] (Table 3), as implemented in the Pombert lab SSRG pipeline (https://github.com/PombertLab).

**Table 3:** Comparison of increases in plasmid copy number and desulfurization specific activity, as well as overall SNPs and indels for CW25[pRESX-*dszAS1BC*] in selection experiments one and three.

Experiment Number	Passage Number	Plasmid:Genome Coverage Ratio	Relative Specific Activity	SNPs (indels) Mapped to P100 Assembly	Total Genome Read Coverage
1	P0	1.2	1.0	37 (3)	43x
	P4	1.9^a^	16	37 (4)	54x
	P100	3.5	3.5	91 (1)	81x
3	P10	4.7	7.2	96 (1)	109x

Relative plasmid copy number was estimated from the ratio of sequence coverage for the plasmid to the sequence coverage for the genome in each sequencing experiment. Relative specific activities are calculated from the data in Figs 4 and 5 with the value for P0 arbitrarily set at 1.0. SNPs and indels are across the entire P100 reference assembly (6,399,510 bp).

^a^ Plasmid coverage indicated the presence of both empty pRESX vector and pRESX-*dszAS1BC*.

## Results

### Transformation of pRESX-dszABC and pRESX-dszAS1BC into strain CW25

**Fig 1:**
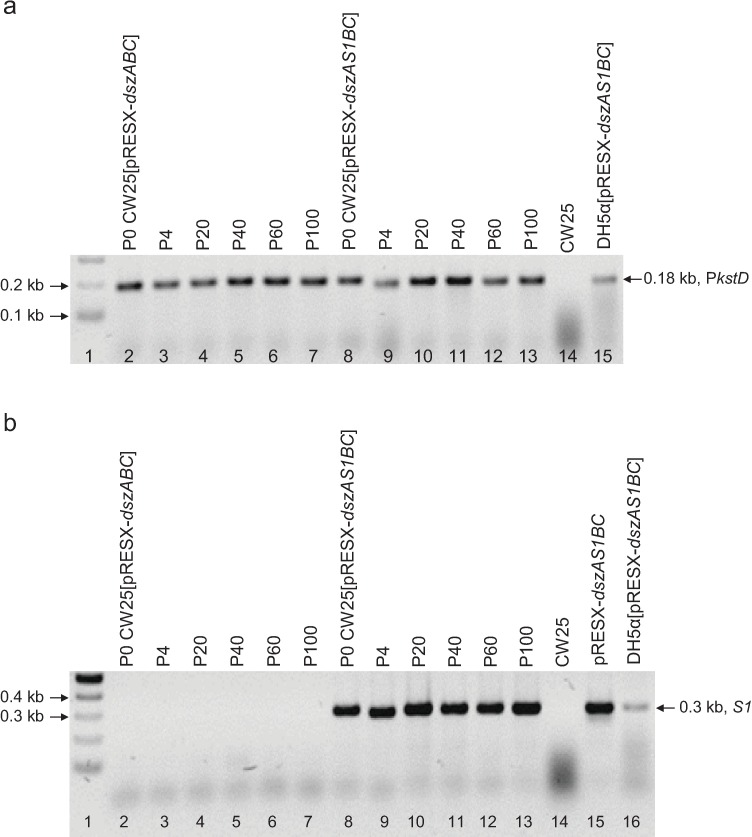
Electrophoretic analysis of plasmid stability in CW25[pRESX-*dszABC*] and CW25[pRESX-*dszAS1BC*] (passages from the first selection experiment). (a) P*kstD* forward and reverse primers were used in colony PCR. Lane 1, 2-log ladder; lanes 2–7, colony PCR of CW25[pRESX-*dszABC*]; lanes 8–13, colony PCR of CW25[pRESX-*dszAS1BC*]; lane 14, colony PCR of CW25 (negative control); lane 15, colony PCR of *E*. *coli* DH5α[pRESX-*dszAS1BC*] (positive control). All amplicons are the expected 0.18 kb. (b) *S1* forward and reverse primers were used in colony PCR. Lane 1, 2-log ladder; lanes 2–7, colony PCR of CW25[pRESX-*dszABC*] (there is, as expected, no amplicon); lanes 8–13, colony PCR of CW25[pRESX-*dszAS1BC*] (amplicons are all of the expected size, 0.3 kb); lane 14, colony PCR of CW25 (negative control); lane 15, PCR of plasmid pRESX-*dszAS1BC* (positive control); lane 16, colony PCR of *E*. *coli* DH5α[pRESX-*dszAS1BC*] (positive control).

Transformants from LB-kanamycin (500 μg/ml) plates were used as templates in colony PCR using P*kstD* primers, diagnostic for the presence of both pRESX-based constructs (Fig 1A), and *S1* primers, diagnostic for the *dszAS1BC* construct (Fig 1B). These results confirmed transformation of CW25 with both pRESX-*dszABC* and pRESX-*dszAS1BC*.

### Sulfur demand and optimal medium for CW25 transformants

**Fig 2:**
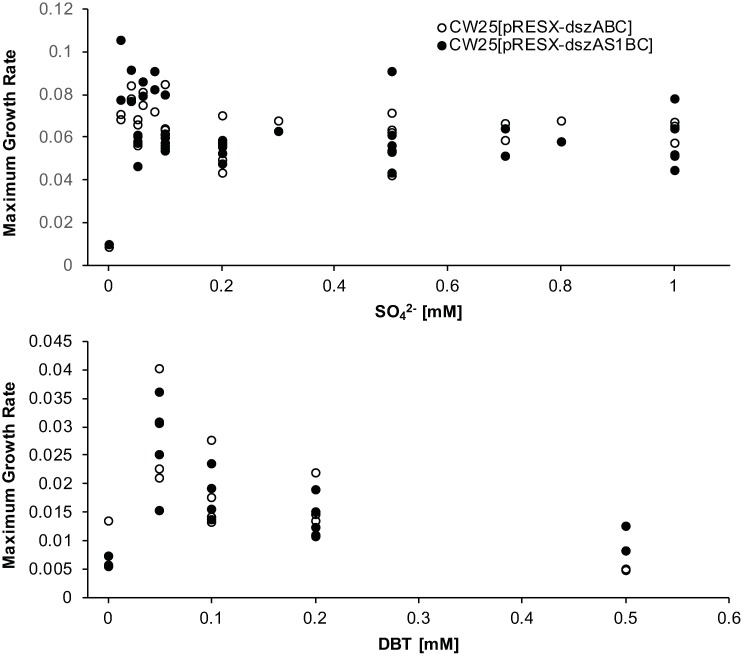
Sulfur demand experiments. Maximum growth rates (generations/hour) of CW25[pRESX-*dszABC*] P0 and CW25[pRESX-*dszAS1BC*] P0 in CDM-SO_4_^2-^ (top panel) and CDM-DBT (bottom panel) media. Each point is an individual determination.

A series of experiments were run to determine the optimal medium for growth and desulfurization activity for the CW25 transformants. Experiments run with varying concentrations of either DBT or sulfate as sole sulfur source showed maximum growth rates at 0.05 mM sulfate or DBT. Higher concentrations of DBT were inhibitory to growth (the actual inhibition perhaps because of higher concentrations of 2-HBP produced [42]), while higher sulfate concentrations neither increased nor decreased growth rates. The maximum growth rate with sulfate was about two to three times that for DBT (Fig 2).

Further experiments measuring desulfurization specific activity of the CW25 transformants identified 0.043 M ethanol as superior to 0.06 M glucose (by about 2-fold) as the carbon source in minimal CDM-DBT. DMSO was inferior to DBT as a sulfur source (by about 5-fold), and 0.1 mM DBT was only slightly superior to 0.05 mM DBT. In addition, as a base minimal medium, CDM was superior to BSM [10] (about 60% better; S1 Fig). Thus, the medium chosen for assessing DszABC specific activity was CDM-0.05 mM DBT containing 0.043 M ethanol as carbon source.

### Growth and specific activity measurements

P0 (the initial transformants; see above), and passages P4, P20, P40, P60 and P100 of both CW25[pRESX-*dszABC*] and CW25[pRESX-*dszAS1BC*] were obtained from the first selection experiment and used as templates in colony PCR using P*kstD* and *S1* primers to confirm the stabilities of pRESX-*dszABC* and pRESX-*dszAS1BC*. The presence of P*kstD* in all passages for both strains (Fig 1A), and *S1* in all passages of only CW25[pRESX-*dszAS1BC*] (Fig 1B) confirmed the stability of both plasmids throughout the entire experiment.

**Fig 3:**
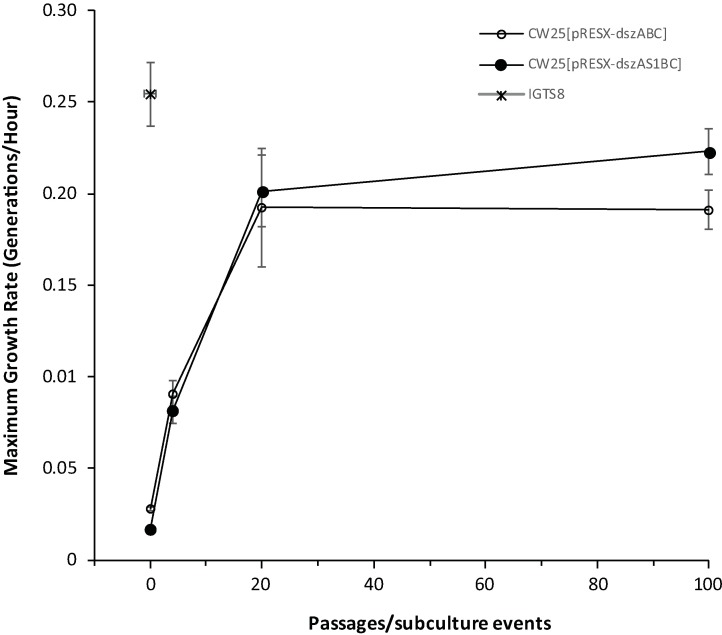
Growth rates of CW25[pRESX-*dszABC*] and CW25[pRESX-*dszAS1BC*] as a function of passage number in the first selection experiment. Passages tested included P0, P4, P20 and P100. IGTS8 was included as a positive control. Values are averages of at least 3 independent measurements (population standard deviations indicated). A one tailed T-test showed that the growth rates for CW25[pRESX-*dszABC*] and CW25[pRESX-*dszAS1BC*] at P100 were significantly different (P value of 0.0064).

As expected from our selection for faster growth, the growth rates of both transformants increased markedly, especially during early passages (Fig 3). Following P20 the growth rate of CW25[pRESX-*dszAS1BC*] was greater than that of CW25[pRESX-*dszABC*]. Overall, from P0 through P100, the growth rates of CW25[pRESX-*dszABC*] and CW25[pRESX-*dszAS1BC*] in medium with DBT as sole sulfur source increased by 6.8-fold and 12.8-fold, respectively.

**Fig 4:**
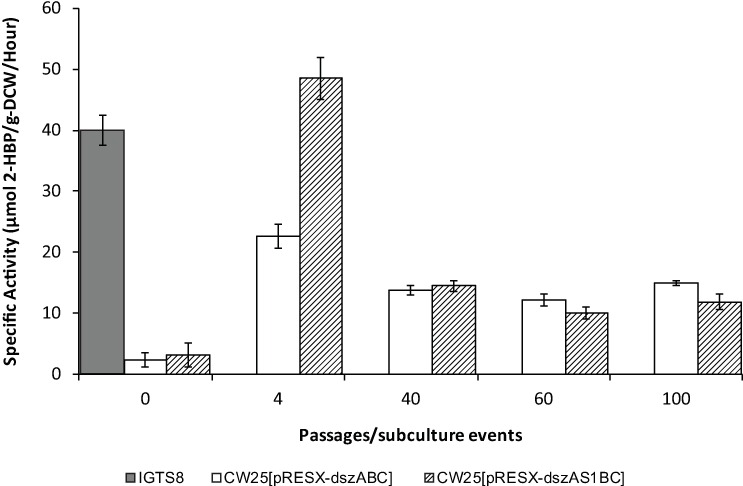
Specific activities of IGTS8 and passages from the first selection experiment. Ethanol (0.043 M) was used as the carbon source in cultures. From left to right: activity of IGTS8 (as control) and passages of CW25[pRESX-*dszABC*] and CW25[pRESX-*dszAS1BC*] including P0, P4, P40, P60 and P100. Values are averages of at least 3 independent determinations (population standard deviations indicated). A one tailed T-test showed that the specific activities for CW25[pRESX-*dszABC*] and CW25[pRESX-*dszAS1BC*] at P4 were significantly different (P value of 0.0011) and those for CW25[pRESX-*dszAS1BC*] and IGTS8 at P4 were significantly different (P value of 0.025).

The same passages, along with IGTS8 as a positive control, were assayed for desulfurization specific activity (Fig 4). The specific activities of P0 CW25[pRESX-*dszABC*] and P0 CW25[pRESX-*dszAS1BC*] were 2.4 units and 3.1 units, respectively, both much lower than IGTS8 (40 units). The specific activities of both were highest at P4 and decreased and stabilized at a constant level (10–16 units) in subsequent passages. The highest specific activity of CW25[pRESX-*dszABC*] was about 23 units, and that of CW25[pRESX-*dszAS1BC*], about 48 units (Fig 4). The specific activity assay was repeated with growth in CDM-0.05 mM DBT with 0.06 M glucose as carbon source with similar results, except that, as expected (see above), activities were generally lower than those of ethanol grown cells (S1 Fig).

**Fig 5:**
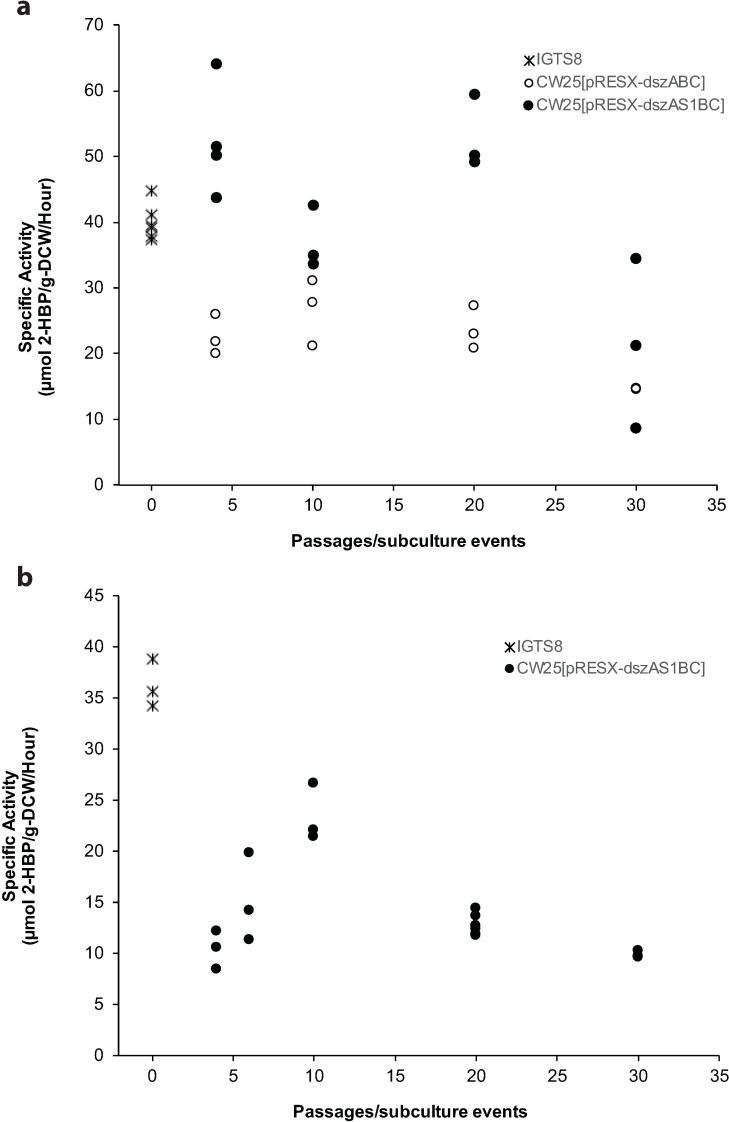
**Specific activities of IGTS8, and CW25[pRESX-*dszABC*] and CW25[pRESX-*dszAS1BC*] passages from the second (a) and third (b) selection experiments as a function of passage number.** Passages included P4, P10, P20 and P30 in (a), and P4, P6, P10, P20 and P30 in (b). Each point is an individual determination.

Two additional selection experiments of 30 passages each were also completed (details in Table 1), and in each case plasmid stability throughout was confirmed by PCR (S2, S3 and S4 Figs), as shown for the first experiment in Fig 1. The results of specific activity measurements for the additional experiments are shown in Fig 5A and 5B. Although there are some differences from the results of the first selection experiment, the overall trends are consistent with them, specifically a gradual decline in specific activity after an early peak. The second experiment also shows that the early specific activity advantage of CW25[pRESX-*dszAS1BC*] over CW25[pRESX-*dszABC*] is substantial through at least 20 passages.

### Overall desulfurization ability

**Fig 6:**
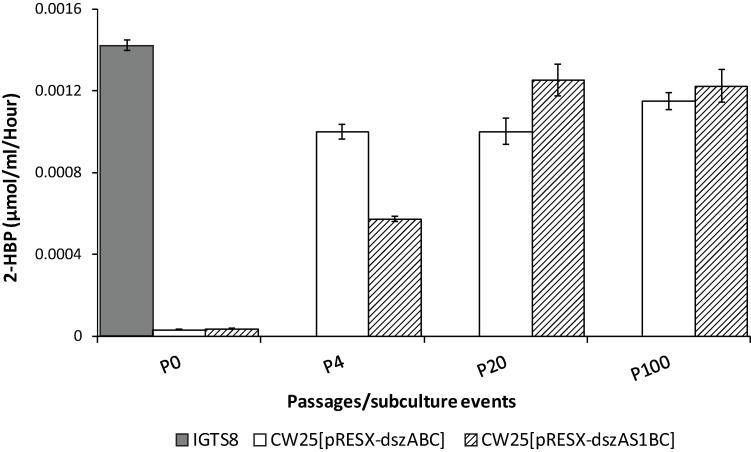
The 2-HBP production rates of growing cells normalized to culture volume. Passages were P0, P4, P20, and P100 from the first selection experiment; IGTS8 was included as a positive control. Ethanol (0.043 M) was used as the carbon source in cultures, and data were taken for cultures at a single point in late log phase. Values are averages of at least 3 independent determinations (population standard deviations indicated). A one tailed T-test showed that the production rates for CW25[pRESX-*dszABC*] and CW25[pRESX-*dszAS1BC*] at P20 were significantly different (P value of 0.0021) and those for CW25[pRESX-*dszABC*] and CW25[pRESX-*dszAS1BC*] at P100 were nearly significantly different (P value of 0.053).

To examine overall desulfurization ability, that due to the combination of growth and specific activity, we examined the rate of accumulation of 2-HBP in the culture broth after growth (in CDM-0.05 mM DBT containing 0.043 M ethanol) of various passages from the first experiment for both strains. Most likely due to the increased growth rates of later passages, the results differ from those of the resting cell specific activity assay. For both transformants, there was a large increase in 2-HBP accumulated per ml of culture to a level that was stable through P100 (36-fold for CW25[pRESX-*dszABC*] and 33-fold for CW25[pRESX-*dszAS1BC*] compared to P0 in each case) (Fig 6). Comparison of these increases with the somewhat smaller increases in specific activity (Fig 4), indicates that the overall increase in DBT metabolized is, not surprisingly, a combination of both better growth and increased specific activity of the later passages. Both transformants eventually performed similarly to IGTS8, the S1 expressing strain doing slightly better than the non S1 expressing strain.

### Whole genome sequencing

The genomes of CW25[pRESX-*dszAS1BC*] passages P0, P4 and P100 from the first experiment and passage P10 from the third experiment were assembled and genes encoding key enzymes in DBT metabolism were identified and compared for the presence of mutations (Table 2). In no case, however, were any mutations found when the starting strain (P0) was compared with P4, P10, and P100. This lack of mutations was not restricted to the Dsz genes; no modification was found in genes involved in the downstream metabolism of sulfites produced by the 4S pathway, in Dam methylases or in the sulfite exporter (TauE/SafE), which would allow the cells to discard sulfite in addition to using it. Homologues to the putative sulfite oxidoreductase identified in *R*. *erythropolis* RP4 [23], sulfite dehydrogenase, or the hemoprotein subunit (CysI) of sulfite reductase were not found in strain CW25. In no case were any of the *dsz* genes transferred from plasmid to genome.

The complete genomes of P0, P4, and P100 from the first selection experiment and P10 from the third selection experiment were also compared to identify all the changes in the genomes that had occurred from P0 to the later passages. The total SNP and indel quantities across all generations were inconsequential, with the largest variation still being less than 0.01 SNPs/kb, and none of the changes occurred in any of the genes we examined. Furthermore, most of the SNPs inferred appear to result from false positives, as all but 3 of the 37 SNPs between P0 and P100 (Table 3) were also found when P100 was mapped against itself. The slightly larger SNP counts inferred for P100 against itself (and P10 against P100) were likely due to an increase in false positives caused by variations in read lengths (post-trimming) and coverage (see genome coverage data in Table 3). The sequence coverage data indicate an increase in plasmid copy number (and thus *dszAS1BC* copy number) as adaptive selection proceeded that fairly closely parallels the increase in desulfurization specific activity (Table 3). Details of the genes in which SNPs were found when the complete genomes of P0 and P100 from the first selection experiment were compared are shown in Table 4. None of the indels found between P0 and P100 were located in coding regions.

**Table 4:** Details of genes in which SNPs were found.

Locus	Type	Status	Product
A3852_00010	Synonymous	false positive	hypothetical protein
A3852_06055	non-synonymous	true SNP	cytochrome C
A3852_18170	non-synonymous	true SNP	non-ribosomal peptide synthetase
A3852_20715	Synonymous + non-synonymous	false positive	non-ribosomal peptide synthetase
A3852_20720	synonymous + non-synonymous	false positive	hypothetical protein
A3852_29435	non-synonymous	true SNP	GntR family transcriptional regulator
A3852_29860	not-applicable	false positive	23S ribosomal RNA

The complete genomes of P0 and P100 from the first selection experiment were compared and the SNPs occurring in P100 (compared to P0) are listed.

## Discussion

The need for cleaner sources of energy requires improvements on many fronts. In the petroleum industry, tighter environmental restrictions dictate for cleaner fuels with ever decreasing amounts of sulfur compounds. While current chemical processes have their own advantages, biodesulfurization may help complement these approaches in a cost effective and environmentally friendly way. To do so, however, biodesulfurizing bacteria will require enzymatic efficiency and thermostability at the temperatures required for petroleum processing [8]. Here, we aimed to improve upon enzymatic efficiency by a combination of genetic engineering and adaptive evolution. This approach resulted in two separate yet integrated effects, an increase in the specific activity of DBT metabolism and a larger increase in growth rate when DBT was the sole sulfur source.

### Desulfurization activity

Intuitively, an enzymatic reaction can be sped up in three ways: by using a faster enzyme, by using more of an enzyme, and/or by preventing the reverse reaction from occurring. Here the improvements observed to the overall biodesulfurization efficiencies of our engineered strains did not result in the use of faster enzymes, as none of the enzymes involved in this process showed any mutation after the maximum number of generations we performed. However, four hundred generations can be considered a short time scale and evolution often occurs by long periods of slow changes interrupted sporadically by quick rapid adaptive bursts, which clearly was not the case here.

What our multiple passages selected for instead is the presence of greater quantities of the enzymes of the desulfurization pathway. The 3.5–7.2X relative increase between the activities of CW25[pRESX-*dszAS1BC*] P0, and P100 of the first selection experiment and P10 of the second selection experiment fairly closely mirrors that of the relative plasmid copy numbers of those passages (3.5–4.7 X) (Table 3). Since none of the *dsz* genes were transferred to the genome and no mutations were found in any chromosomal genes involved in plasmid replication, this increase in copy number clearly originates from the plasmid itself.

After the initial increases in specific activities in the early passages, all three replicates stabilized at roughly the same levels, suggesting that four plasmids per bacterium may be the maximum sustainable for both the S1-bearing and S1-less strains under the conditions used in this study. The high specific activity levels of the sulpeptide expressing strain in early passages of the first and second experiments were actually greater than those of IGTS8. Although transient, these increases could possibly be related to physiological changes occurring during the selection process (see also below).

When planning this experiment, we hoped to use the sulpeptide as a sulfur sink that would enhance the metabolism of DBT by accumulating sulfur released by the 4S pathway. However, except for the early passages in selection experiments 1 and 2 (Figs 4 and 5A) in which the specific activity of the sulpeptide-plus transformant was substantially greater than that of the sulpeptide-minus transformant, the increases in specific activity of the two strains eventually stabilized at levels that were generally similar to each other. Possible causes for this could be that the enzymes are already performing at maximum efficiency or that the reverse reaction is so slow that the removal of sulfite produced by the 4S pathway by the sulpeptide would have a negligible effect on the net reaction.

Another possibility is that the sulfur sink produced by the sulpeptide may be too small to have a substantial effect on sulfur demand. Specifically, the 14 methionines and cysteines encoded in S1 constitute but a tiny fraction compared to the total of 43,754 that are encoded in proteins from the main chromosome, and unless the plasmid expression level surpasses that of the genome by two or more orders of magnitude, the net effect from S1 alone is likely to be negligible (although the combined effect of all the proteins encoded on the plasmid might have an impact at a much lower threshold). Attempts to quantify S1 production using SDS gel electrophoresis were unsuccessful because of the small size of S1 and, perhaps, low levels of its synthesis. In any case, from these results we can conclude that increasing the number of *dsz* genes in the bacterium either by using a plasmid as shown here or by integration into the main chromosome yields greater desulfurization benefits and should be a key engineering focus.

### Growth rate and cumulative 2-HBP production during growth

Because we selected for the fastest growing cells in each passage, it is not surprising that the growth rates of both strains increased greatly as the number of passages increased. During the first selection experiment, about 90% of the increases occurred in the first 20 passages, suggesting that the maximum growth rates for these conditions had been approached by that time (Fig 3); these rates were similar to that of IGTS8, even though occurring in a strain with no inherent DBT metabolizing ability. The overall pattern of the increase in growth rates was similar for both strains and at least qualitatively paralleled the patterns of both 4S pathway specific activity (Fig 4) and overall DBT metabolism (Fig 6) (see also below). This is as expected since growth is dependent on sulfur obtained from DBT via the 4S pathway

While the presence of S1 did not affect the eventual specific desulfurization activity, its addition did have a positive effect on the growth rate (about twice the overall improvement and 17% faster maximal growth compared to the strain lacking sulpeptide (Fig 3)). The net effect was a small advantage in overall biodesulfurization ability of the sulpeptide-bearing strain (Fig 6). In any case, the selection process itself, apart from the influence of S1, was successful in producing large and stable improvements in overall desulfurization.

The maximum growth rates of both strains with DBT as sole sulfur source (at P100 of the first selection experiment) were greater (by about two to three times) than those of the respective P0 strains grown with excess sulfate as sole sulfur source. However, what causes this higher growth rate is unclear. In the sulpeptide-containing strain, only three insertion/deletions (indels) and 37 single nucleotide polymorphisms (SNPs) were found after about 400 generations between the P0 and P100 isolates, none of which were located in sulfur-related genes or promoters. While SNPs located in the 23S ribosomal RNA gene (A3852_29860; Table 4) could in theory affect a wide spectrum of functions via its role in translation, and thus potentially explain the observed changes in growth rate (a more efficient ribosome, even if only slightly, would certainly reap tangible benefits), the inferred mutations were not genuine and resulted from erroneous read mapping. The 23S ribosomal RNA gene is present in multiple copies in the genome, and SNPs inferred when duplicate or paralogous genes are involved tend to be overestimated due to the inherent limitations of read mapping-based polymorphism inferences [43].

On the other hand, the single mutation observed in the transcriptional regulator of the GntR family (A3852_29435) appears genuine, and the non-synonymous change from a hydrophilic residue (Ser-215) to a hydrophobic one (Gly) in P100 could have implications on its overall fitness, and thus potentially affect the expression of genes regulated by this factor. Non-synonymous changes between P0 and P100 were also observed in genes coding for cytochrome C and a non-ribosomal peptide synthetase (Table 4), but the effects that such mutations could have on the fitness of the bacterium is unclear.

Nevertheless, considering that both the sulpeptide-bearing and sulpeptide-minus strains independently achieved greater growth rates at similar thresholds, under the same conditions, we do not believe that genetic mutations are behind this adaptation. It would be very unlikely for independent strains to achieve the same level of fitness via random mutagenesis, especially over such a small evolutionary time scale. Changes in gene expression not due to mutation (as observed in long term adaptation studies of *Yersinia pestis* [44]) are targets for future investigation (for example by proteomic studies); such changes may occur via heritable epigenetic changes [45], changes in regulatory circuits [46], or combinations of these mechanisms. Heritable epigenetic changes, sometimes induced by environmental factors, have been documented in a number of cases, mediated by the Dam methylase or similar enzymes [45]. If such mechanisms are involved in our system, however, they cannot be due to mutations to the Dam methylase gene in strain CW25, as no changes were found in its sequence in any of the four passages examined.

It is also possible that the changes in growth and metabolism we saw in the various passages may be due to physiological adaptations [46,47]. Such adaptations could be maintained by changes in the levels of transcriptional regulators, responding to environmental conditions, such as nutrient limitation. In Gram-negative *E*. *coli*, such changes are mediated by the RpoS sigma factor [46,47], while in Gram positive *C*. *glutamicum* and *M*. *tuberculosis* the SigB sigma factor plays a similar role [48,49]. In the experiments described here, limiting the sulfur source to a sparingly soluble molecule like DBT could be a signal of nutrient limitation. Any adaptations due to changes in functions of the alternative sigma factor SigB in *Rhodococcus* CW25, however, also cannot be due to mutations in the *sigB* gene, which did not occur in any of the later passages examined.

Taken together the results suggest that further work to increase biodesulfurization will have to take a global approach, considering a combination of genetic, epigenetic, and physiological changes. In addition, a better experimental strategy might be to first select a culture for maximum growth rate using sulfate, and then use this physiologically tuned culture as the starting point for selection for maximum growth rate with DBT. In this way changes in overall metabolic fitness optimization would have less chance of overshadowing changes that are specific to DBT metabolism.

## Conclusions

Engineering of desulfurization competent *Rhodococcus qingshengii* to express a peptide designed to act as a sulfur sink (Sulpeptide 1), combined with adaptive evolution over the course of about 400 generations was successful in substantially increasing both their metabolism of DBT and their growth rate in medium with DBT as the sole sulfur source. The increased growth rate on DBT eventually exceeded (by more than 2-fold) the growth rate of unadapted strains grown with sulfate as sole sulfur source. The effect of the expression of Sulpeptide 1 contributed less to these increases than did the adaptive evolution.

The increases in growth rate and DBT metabolism were not correlated to any mutations in genes of obvious connection to DBT metabolism or sulfur metabolism in general, although the eventual increase in DBT metabolism was closely correlated to an increase in copy number of the genes encoding DBT metabolism. It may be that the important changes that occurred during the adaptation may include epigenetic and stable physiological ones. These results suggest that future research should first optimize growth on sulfate under ideal conditions and only then should the *dsz* genes be introduced and adaptive evolution experiments be performed.

## Supporting Information

S1 Fig Optimization of growth medium for CW25 derivatives regarding 4S pathway specific activity (units of μmoles 2-HBP produced per gram dry cell weight of cells per hour). (a) Comparison of different carbon sources with different concentrations. (b) Comparison of different sulfur sources with different concentrations; for the DMSO/DBT induction experiment, 0.05 mM DBT was added to induce Dsz activity of the culture grown on DMSO 5 hours prior to harvest. (c) Comparison of CDM and BSM media. Values are averages of at least 3 independent determinations (population standard deviations indicated). In these experiments, P40 of CW25[pRESX-*dszAS1BC*] from the first selection experiment was used.
(DOCX)

S2 Fig Amplification of P*kstD* from CW25[pRESX-*dszABC*] and CW25[pRESX-*dszAS1BC*] passages from the second selection experiment. Passages included P10, P20, and P30. Lanes 1, 9 and 23, 2-log ladder; lanes 2 and 10, negative control without template. Three different CW25[pRESX-*dszABC*] colonies selected from P10 are in lanes 3–5, from P20 are in lanes 11–13, and from P30 are in lanes 14–16. Three different CW25[pRESX-*dszAS1BC*] colonies selected from P10 are in lanes 6–8, from P20 are in lanes 17–19, and from P30 are in lanes 20–22. A fragment of the size expected for the P*kstD* promoter (0.18 kb) was amplified from all samples.
(DOCX)

S3 Fig Amplification of *S1* from CW25[pRESX-*dszABC*] and CW25[pRESX-*dszAS1BC*] passages from the second selection experiment. Passages included P10, P20 and P30. Lanes 1 and 9, 2-log ladder; lanes 2 and 10, negative control without template. Three different CW25[pRESX-*dszABC*] colonies selected from P10 are in lanes 3–5, from P20 are in lanes 11–13, and from P30 are in lanes 14–16. Three different CW25[pRESX-*dszAS1BC*] colonies selected from P10 are in lanes 6–8, from P20 are in lanes 17–19, and from P30 are in lanes 20–22. A fragment of the size expected for *S1* (0.3 kb) was amplified only from CW25[pRESX-*dszAS1BC*] samples.
(DOCX)

S4 Fig Amplification of *S1* from CW25[pRESX-*dszAS1BC*] passages from the third selection experiment. Passages included P4, P6, P10, P20 and P30. Lanes 1 and 6, 2-log ladder; lane 17, HindIII digested lambda DNA ladder; lanes 2, 7, and 18, negative control without template. Three different CW25[pRESX-*dszAS1BC*] colonies selected from P4 are in lanes 3–5, from P6 are in lanes 8–10, from P10 are in lanes 11–13, from P20 are in lanes 19–21, and from P30 are in lanes 14–16. A fragment of the size expected for *S1* (0.3 kb) was amplified from all samples.
(DOCX)

## References

[1] Kilbane JJ (1989) Desulfurization of coal: the microbial solution. Trends Biotechnol 7: 97–101.

[2] Brunet S, Mey D, Pérot G, Bouchy C, Diehl F (2005) On the hydrodesulfurization of FCC gasoline: a review. Appl Catal A Gen 278: 143–172.

[3] Qiu L, Cheng Y, Yang C, Zeng G, Long Z, Wei S, et al (2016) Oxidative desulfurization of dibenzothiophene using a catalyst of molybdenum supported on modified medicinal stone. RSC Adv 6: 17036–17045.

[4] Yang C, Zhao K, Cheng Y, Zeng G, Zhang M, Shao J, et al (2016) Catalytic oxidative desulfurization of BT and DBT from n-octane using cyclohexanone peroxide and catalyst of molybdenum supported on 4A molecular sieve. Sep Purif Technol 163: 153–161.

[5] Zhao K, Cheng Y, Liu H, Yang C, Qiu L, Zeng G, et al (2015) Extractive desulfurization of dibenzothiophene by a mixed extractant of N,N-dimethylacetamide, N,N-dimethylformamide and tetramethylene sulfone: optimization by Box-Behnken design. RSC Adv 5: 66013–66023.

[6] Corro G (2002) Sulfur impact on diesel emission control—A review. React Kinet Catal Lett 75: 89–106.

[7] McFarland BL, Boron DJ, Deever W, Meyer JA, Johnson AR, Atlas RM (1998) Biocatalytic sulfur removal from fuels: applicability for producing low sulfur gasoline. Crit Rev Microbiol 24: 99–147. 10.1080/10408419891294208 9675512

[8] Kilbane JJ (2006) Microbial biocatalyst developments to upgrade fossil fuels. Curr Opin Biotechnol 17: 305–314. 10.1016/j.copbio.2006.04.005 16678400

[9] Kilbane JJ, Jackowski K (1992) Biodesulfurization of water-soluble coal-derived material by *Rhodococcus rhodochrous* IGTS8. Biotechnol Bioeng 40: 1107–1114. 10.1002/bit.260400915 18601220

[10] Denome SA, Oldfield C, Nash LJ, Young KD (1994) Characterization of the desulfurization genes from *Rhodococcus* sp. strain IGTS8. J Bacteriol 176: 6707–6716. 7961424 10.1128/jb.176.21.6707-6716.1994 PMC197028

[11] Lei B, Tu SC (1996) Gene overexpression, purification, and identification of a desulfurization enzyme from *Rhodococcus* sp. strain IGTS8 as a sulfide/sulfoxide monooxygenase. J Bacteriol 178: 5699–5705. 8824615 10.1128/jb.178.19.5699-5705.1996 PMC178409

[12] Gray KA, Pogrebinsky OS, Mrachko GT, Xi L, Monticello DJ, Squires CH (1996) Molecular mechanisms of biocatalytic desulfurization of fossil fuels. Nat Biotechnol 14: 1705–1709. 10.1038/nbt1296-1705 9634856

[13] Gray KA, Mrachko GT, Squires CH (2003) Biodesulfurization of fossil fuels. Curr Opin Microbiol 6: 229–235. 12831898 10.1016/s1369-5274(03)00065-1

[14] Kilbane JJ, Le Borgne S (2004) Chapter 2 Petroleum biorefining: the selective removal of sulfur, nitrogen, and metals. Stud Surf Sci Catal 151: 29–65.

[15] Konishi J, Ishii Y, Onaka T, Okumura K, Suzuki M (1997) Thermophilic carbon-sulfur-bond-targeted biodesulfurization. Appl Environ Microbiol 63: 3164–3169. 16535672 10.1128/aem.63.8.3164-3169.1997 PMC1389227

[16] Ishii Y, Konishi J, Okada H, Hirasawa K, Onaka T, Suzuki M (2000) Operon structure and functional analysis of the genes encoding thermophilic desulfurizing enzymes of *Paenibacillus* sp. A11-2. Biochem Biophys Res Commun 270: 81–88. 10.1006/bbrc.2000.2370 10733908

[17] Kirimura K, Furuya T, Nishii Y, Ishii Y, Kino K, Usami S (2001) Biodesulfurization of dibenzothiophene and its derivatives through the selective cleavage of carbon-sulfur bonds by a moderately thermophilic bacterium *Bacillus subtilis* WU-S2B. J Biosci Bioeng 91: 262–266. 16232986 10.1263/jbb.91.262

[18] Furuya T, Kirimura K, Kino K, Usami S (2001) Thermophilic biodesulfurization of dibenzothiophene and its derivatives by *Mycobacterium phlei* WU-F1. FEMS Microbiol Lett 204: 129–133. 11682191 10.1111/j.1574-6968.2001.tb10875.x

[19] Li FL, Xu P, Ma CQ, Luo LL, Wang XS (2003) Deep desulfurization of hydrodesulfurization-treated diesel oil by a facultative thermophilic bacterium *Mycobacterium* sp. X7B. FEMS Microbiol Lett 223: 301–307. 12829302 10.1016/S0378-1097(03)00397-5

[20] Kayser KJ, Cleveland L, Park H-S, Kwak J-H, Kolhatkar A, Kilbane JJ II (2002) Isolation and characterization of a moderate thermophile, *Mycobacterium phlei* GTIS10, capable of dibenzothiophene desulfurization. Appl Microbiol Biotechnol 59: 737–745. 10.1007/s00253-002-1030-8 12226734

[21] Hirasawa K, Ishii Y, Kobayashi M, Koizumi K, Maruhashi K (2001) Improvement of desulfurization activity in *Rhodococcus erythropolis* KA2-5-1 by genetic engineering. Biosci Biotechnol Biochem 65: 239–246. 11302154 10.1271/bbb.65.239

[22] Li G-Q, Ma T, Li S-S, Li H, Liang F-L, Liu R-L (2007) Improvement of dibenzothiophene desulfurization activity by removing the gene overlap in the *dsz* operon. Biosci Biotechnol Biochem 71: 849–854. 10.1271/bbb.60189 17420595

[23] Aggarwal S, Karimi IA, Kilbane II JJ, Lee DY (2012) Roles of sulfite oxidoreductase and sulfite reductase in improving desulfurization by *Rhodococcus erythropolis*. Mol Biosyst 8: 2724–2732. 10.1039/c2mb25127b 22832889

[24] Pan J, Wu F, Wang J, Xu L, Khayyat NHN, Stark BC, et al (2013) Enhancement of desulfurization activity by enzymes of the *Rhodococcus dsz* operon through coexpression of a high sulfur peptide and directed evolution. Fuel 112: 385–390.

[25] van der Geize R, Hessels GI, Nienhuis-Kuiper M, Dijkhuizen L (2008) Characterization of a second *Rhodococcus erythropolis* SQ1 3-ketosteroid 9 hydroxylase activity comprising a terminal oxygenase homologue, KshA2, active with oxygenase-reductase component KshB. Appl Environ Microbiol 74: 7197–7203. 10.1128/AEM.00888-08 18836008 PMC2592919

[26] Wang J, Davaadelger B, Salazar JK, Butler RR, Pombert J-F, Kilbane JJ, et al (2015) Isolation and characterization of an interactive culture of two *Paenibacillus* species with moderately thermophilic desulfurization ability. Biotechnol Lett 37: 2201–2211. 10.1007/s10529-015-1918-x 26209032

[27] Pearlman RS, Yalkowsky SH, Banerjee S (1984) Water solubilities of polynuclear aromatic and heteroaromatic compounds. J Phys Chem Ref Data 13: 555.

[28] Hanahan D (1983) Studies on transformation of *Escherichia coli* with plasmids. J Mol Biol 166: 557–580. 6345791 10.1016/s0022-2836(83)80284-8

[29] Bankevich A, Nurk S, Antipov D, Gurevich AA, Dvorkin M, Kulikov AS, et al (2012) SPAdes: a new genome assembly algorithm and its applications to single-cell sequencing. J Comput Biol 19: 455–477. 10.1089/cmb.2012.0021 22506599 PMC3342519

[30] Boisvert S, Raymond F, Godzaridis E, Laviolette F, Corbeil J (2012) Ray Meta: scalable *de novo* metagenome assembly and profiling. Genome Biol 13: R122 10.1186/gb-2012-13-12-r122 23259615 PMC4056372

[31] Coil D, Jospin G, Darling AE (2015) A5-miseq: an updated pipeline to assemble microbial genomes from Illumina MiSeq data. Bioinformatics 31: 587–589. 10.1093/bioinformatics/btu661 25338718

[32] Kearse M, Moir R, Wilson A, Stones-Havas S, Cheung M, Sturrock S, et al (2012) Geneious Basic: an integrated and extendable desktop software platform for the organization and analysis of sequence data. Bioinformatics 28: 1647–1649. 10.1093/bioinformatics/bts199 22543367 PMC3371832

[33] Chikhi R, Medvedev P (2014) Informed and automated k-mer size selection for genome assembly. Bioinformatics 30: 31–37. 10.1093/bioinformatics/btt310 23732276

[34] Gurevich A, Saveliev V, Vyahhi N, Tesler G (2013) QUAST: quality assessment tool for genome assemblies. Bioinformatics 29: 1072–1075. 10.1093/bioinformatics/btt086 23422339 PMC3624806

[35] Seemann T (2014) Prokka: rapid prokaryotic genome annotation. Bioinformatics 30: 2068–2069. 10.1093/bioinformatics/btu153 24642063

[36] Tatusova T, Ciufo S, Fedorov B, O’Neill K, Tolstoy I (2014) RefSeq microbial genomes database: new representation and annotation strategy. Nucleic Acids Res 42: D553–9. 10.1093/nar/gkt1274 24316578 PMC3965038

[37] Altschul SF, Gish W, Miller W, Myers EW, Lipman DJ (1990) Basic Local Alignment Search Tool. J Mol Biol 215: 403–410. 10.1016/S0022-2836(05)80360-2 2231712

[38] Caspi R, Billington R, Ferrer L, Foerster H, Fulcher CA, Keseler IM, et al (2016) The MetaCyc database of metabolic pathways and enzymes and the BioCyc collection of pathway/genome databases. Nucleic Acids Res 44: D471–80. 10.1093/nar/gkv1164 26527732 PMC4702838

[39] Li H, Durbin R (2010) Fast and accurate long-read alignment with Burrows-Wheeler transform. Bioinformatics 26: 589–595. 10.1093/bioinformatics/btp698 20080505 PMC2828108

[40] Li H, Handsaker B, Wysoker A, Fennell T, Ruan J, Homer N, et al (2009) The Sequence Alignment/Map format and SAMtools. Bioinformatics 25: 2078–2079. 10.1093/bioinformatics/btp352 19505943 PMC2723002

[41] Koboldt DC, Zhang Q, Larson DE, Shen D, McLellan MD, Lin L, et al (2012) VarScan 2: somatic mutation and copy number alteration discovery in cancer by exome sequencing. Genome Res 22: 568–576. 10.1101/gr.129684.111 22300766 PMC3290792

[42] Abin-Fuentes A, Mohamed ME-S, Wang DIC, Prather KLJ (2013) Exploring the mechanism of biocatalyst inhibition in microbial desulfurization. Appl Environ Microbiol 79: 7807–7817. 10.1128/AEM.02696-13 24096431 PMC3837836

[43] Subramanian S, Di Pierro V, Shah H, Jayaprakash AD, Weisberger I, Shim J, et al (2013) MiST: a new approach to variant detection in deep sequencing datasets. Nucleic Acids Res 41: e154 10.1093/nar/gkt551 23828039 PMC3763541

[44] Leiser OP, Merkley ED, Clowers BH, Deatherage Kaiser BL, Lin A, Hutchison JR, et al (2015) Investigation of *Yersinia pestis* laboratory adaptation through a combined genomics and proteomics approach. PLoS One 10: e0142997 10.1371/journal.pone.0142997 26599979 PMC4658026

[45] Casadesús J, Low D (2006) Epigenetic gene regulation in the bacterial world. Microbiol Mol Biol Rev 70: 830–856. 10.1128/MMBR.00016-06 16959970 PMC1594586

[46] Ferenci T (2005) Maintaining a healthy SPANC balance through regulatory and mutational adaptation. Mol Microbiol 57: 1–8. 10.1111/j.1365-2958.2005.04649.x 15948944

[47] Ryall B, Eydallin G, Ferenci T (2012) Culture history and population heterogeneity as determinants of bacterial adaptation: the adaptomics of a single environmental transition. Microbiol Mol Biol Rev 76: 597–625. 10.1128/MMBR.05028-11 22933562 PMC3429624

[48] Larisch C, Nakunst D, Hüser AT, Tauch A, Kalinowski J (2007) The alternative sigma factor SigB of *Corynebacterium glutamicum* modulates global gene expression during transition from exponential growth to stationary phase. BMC Genomics 8: 4 10.1186/1471-2164-8-4 17204139 PMC1779776

[49] Hu Y, Coates AR (1999) Transcription of two sigma 70 homologue genes, *sigA* and *sigB*, in stationary-phase *Mycobacterium tuberculosis*. J Bacteriol 181: 469–476. 9882660 10.1128/jb.181.2.469-476.1999 PMC93400

