# Supplementary material for: Enhancement of Microbial Biodesulfurization via Genetic Engineering and Adaptive Evolution
Source: PLoS One. 2017 Jan 6;12(1):e0168833. doi: 10.1371/journal.pone.0168833 (PMC5218467; doi:10.1371/journal.pone.0168833)
Supplement: S2 Fig — Passages included P10, P20, and P30. Lanes 1, 9 and 23, 2-log ladder; lanes 2 and 10, negative control without template. Three different CW25[pRESX-dszABC] colonies selected from P10 are in lanes 3–5, from P20 are in lanes 11–13, and from P30 are in lanes 14–16. Three different CW25[pRESX-dszAS1BC] colonies selected from P10 are in lanes 6–8, from P20 are in lanes 17–19, and from P30 are in lanes 20–22. A fragment of the size expected for the PkstD promoter (0.18 kb) was amplified from all samples. (DOCX) [file pone.0168833.s002.docx]

P30

P30

P30 CW25[pRESX-*dszAS1BC*]

P20

P20

P20 CW25[pRESX-*dszAS1BC*]

P30

P30

P30 CW25[pRESX-*dszABC*]

P20

P20

P20 CW25[pRESX-*dszABC*]

Negative control

P10

P10

P10 CW25[pRESX-*dszAS1BC*]

P10

P10

P10 CW25[pRESX-*dszABC*]

Negative control


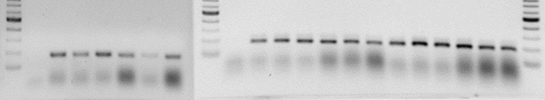


0.18 kb, P*kstD*

0.1 kb

0.2 kb

1 2 3 4 5 6 7 8 9 10 11 12 13 14 15 16 17 18 19 20 21 22 23
