# Supplementary material for: Enhancement of Microbial Biodesulfurization via Genetic Engineering and Adaptive Evolution
Source: PLoS One. 2017 Jan 6;12(1):e0168833. doi: 10.1371/journal.pone.0168833 (PMC5218467; doi:10.1371/journal.pone.0168833)
Supplement: S3 Fig — Passages included P10, P20 and P30. Lanes 1 and 9, 2-log ladder; lanes 2 and 10, negative control without template. Three different CW25[pRESX-dszABC] colonies selected from P10 are in lanes 3–5, from P20 are in lanes 11–13, and from P30 are in lanes 14–16. Three different CW25[pRESX-dszAS1BC] colonies selected from P10 are in lanes 6–8, from P20 are in lanes 17–19, and from P30 are in lanes 20–22. A fragment of the size expected for S1 (0.3 kb) was amplified only from CW25[pRESX-dszAS1BC] samples. (DOCX) [file pone.0168833.s003.docx]

Negative control

P30

P30

P30 CW25[pRESX-*dszAS1BC*]

P20 CW25[pRESX-*dszAS1BC*]

P20

P20

P30

P30

P30 CW25[pRESX-*dszABC*]

P20

P20

P20 CW25[pRESX-*dszABC*]

P10

P10

P10 CW25[pRESX-*dszAS1BC*]

P10

P10

P10 CW25[pRESX-*dszABC*]

Negative control

0.3 kb

0.3 kb, *S1*


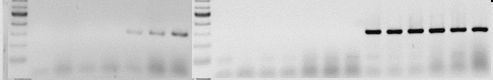


1 2 3 4 5 6 7 8 9 10 11 12 13 14 15 16 17 18 19 20 21 22
