# Supplementary material for: Enhancement of Microbial Biodesulfurization via Genetic Engineering and Adaptive Evolution
Source: PLoS One. 2017 Jan 6;12(1):e0168833. doi: 10.1371/journal.pone.0168833 (PMC5218467; doi:10.1371/journal.pone.0168833)
Supplement: S4 Fig — Passages included P4, P6, P10, P20 and P30. Lanes 1 and 6, 2-log ladder; lane 17, HindIII digested lambda DNA ladder; lanes 2, 7, and 18, negative control without template. Three different CW25[pRESX-dszAS1BC] colonies selected from P4 are in lanes 3–5, from P6 are in lanes 8–10, from P10 are in lanes 11–13, from P20 are in lanes 19–21, and from P30 are in lanes 14–16. A fragment of the size expected for S1 (0.3 kb) was amplified from all samples. (DOCX) [file pone.0168833.s004.docx]

P4

Negative control

P30 CW25[pRESX-*dszAS1BC*]

P30

P30

P10

P10

P10 CW25[pRESX-*dszAS1BC*]

Negative control

P6 CW25[pRESX-*dszAS1BC*]

P6

P6

P4 CW25[pRESX-*dszAS1BC*]

P4


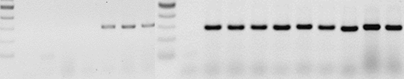


0.3 kb

0.4 kb

3 1 2 3 4 5 6 7 8 9 10 11 12 13 14 15 16

0.3 kb

P20

P20

P20 CW25[pRESX-*dszAS1BC*]

Negative control


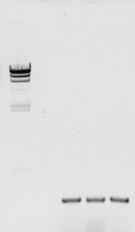


0.3 kb, *S1*

0.6 kb

3 1 17 18 19 20 21
